# Association of total energy intake and macronutrient consumption with colorectal cancer risk: results from a large population-based case-control study in Newfoundland and Labrador and Ontario, Canada

**DOI:** 10.1186/1475-2891-11-18

**Published:** 2012-03-26

**Authors:** Zhuoyu Sun, Lin Liu, Peizhong Peter Wang, Barbara Roebothan, Jin Zhao, Elizabeth Dicks, Michelle Cotterchio, Sharon Buehler, Peter T Campbell, John R Mclaughlin, Patrick S Parfrey

**Affiliations:** 1 Division of Community Health and Humanities, Faculty of Medicine, Memorial University of Newfoundland, St. John's, NL, Canada; 2 School of Public Health, Tianjin Medical University, Tianjin, China; 3 First Affiliated Hospital, Wenzhou Medical College, Wenzhou, China; 4 Clinical Epidemiology Unit, Memorial University of Newfoundland, St. John's, NL, Canada; 5 Population Study and Surveillance, Cancer Care Ontario, Toronto, ON, Canada; 6 Epidemiology Research Program, American Cancer Society, Atlanta, GA, USA

**Keywords:** Colorectal cancer, Total energy, Macronutrient, Case-control study

## Abstract

**Background:**

Diet is regarded as one of the most important environmental factors associated with colorectal cancer (CRC) risk. A recent report comprehensively concluded that total energy intake does not have a simple relationship with CRC risk, and that the data were inconsistent for carbohydrate, cholesterol and protein. The objective of this study was to identify the associations of CRC risk with dietary intakes of total energy, protein, fat, carbohydrate, fiber, and alcohol using data from a large case-control study conducted in Newfoundland and Labrador (NL) and Ontario (ON), Canada.

**Methods:**

Incident colorectal cancer cases (n = 1760) were identified from population-based cancer registries in the provinces of ON (1997-2000) and NL (1999-2003). Controls (n = 2481) were a random sample of residents in each province, aged 20-74 years. Family history questionnaire (FHQ), personal history questionnaire (PHQ), and food frequency questionnaire (FFQ) were used to collect study data. Logistic regression was used to evaluate the association of intakes of total energy, macronutrients and alcohol with CRC risk.

**Results:**

Total energy intake was associated with higher risk of CRC (OR: 1.56; 95% CI: 1.21-2.01, *p*-trend = 0.02, 5^th ^versus 1^st ^quintile), whereas inverse associations emerged for intakes of protein (OR: 0.85, 95%CI: 0.69-1.00, *p*-trend = 0.06, 5^th ^versus 1^st ^quintile), carbohydrate (OR: 0.81, 95%CI: 0.63-1.00, *p*-trend = 0.05, 5^th ^versus 1^st ^quintile) and total dietary fiber (OR: 0.84, 95% CI:0.67-0.99, *p*-trend = 0.04, 5^th ^versus 1^st ^quintile). Total fat, alcohol, saturated fatty acids, monounsaturated fatty acids, polyunsaturated fatty acids, and cholesterol were not associated with CRC risk.

**Conclusion:**

This study provides further evidence that high energy intake may increase risk of incident CRC, whereas diets high in protein, fiber, and carbohydrate may reduce the risk of the disease.

## Introduction

In Canada, colorectal cancer (CRC) is the second leading cause of death from cancer in men and women combined. In 2011, an estimated 22,200 Canadians will be diagnosed with colorectal cancer and 8,900 will die from it [1]. Genetics research found that high-penetrance mutations account for a small proportion of all CRC cases, with low-penetrance mutations accounting for much of the predisposition, which indicated that 'sporadic' CRC contributed to a large amount of CRC-associated morbidity. [2]. Immigrants rapidly acquire the incidence rates of the host country, suggesting that environmental factors play a crucial role in CRC development [3,4].

Diet is regarded as one of the most important environmental factors associated with CRC risk [5,6], particularly when diet is considered in the context of other energy balance indicators such as body size, physical activity, and alcohol or tobacco intake [7-10]. Several case- control studies have demonstrated a higher risk of colorectal cancer with increased total energy intake [9,11-15]. Thus, excess intake of any of the important energy-supplying macronutrients in the diet (e.g., protein, fat and carbohydrate) could contribute to a higher risk of CRC. However, the question of whether or not individual energy-supplying macronutrients, independent of their contribution to energy intake, are related to CRC risk remains controversial.

A Swiss case-control study claimed that energy intake is directly related to colorectal cancer risk, and that different types of fat may have different roles in colorectal carcinogenesis [16]. Specifically, the risk of CRC increased with total energy intake: consumption of saturated fats appeared a borderline significant relation to CRC risk, while monounsaturated and polyunsaturated fats showed significant inverse trends. In contrast, another case-control study reported that intakes of total fat, saturated fat, and monounsaturated fat were not related to risk of colon cancer, whereas the percent of energy from protein was associated with a risk reduction [14]. Non-red meat sources of animal protein, often derived from low-fat dairy products, fish and poultry sources, have been typically associated with lower risks of CRC [17-20]. A recent comprehensive report from the World Cancer Research Fund and the American Institute for Cancer Research concluded that total energy intake has no simple relationship with CRC risk, but its effect may be dependent on other factors, such as physical activity. As well, that data were inconsistent for carbohydrates, cholesterol and proteins [21].

Given the high incidence rate of CRC in Newfoundland and Labrador (NL) and Ontario (ON), it is of great public health importance to identify modifiable risk factors for CRC, including diet. We investigated the associations of total energy intake, macronutrient intake and alcohol in data from a large population based case-control study conducted in NL and ON.

## Materials and methods

### Study population and data collection

The selection progress of cases and controls and study design have been reported previously [22]. Briefly, this case-control study involved 3998 participants from ON and 1420 subjects from NL. Eligibility criteria included people between 20 and 74 year of age, residents of ON and NL. Province cancer registries in ON and NL were used to identify and confirm case eligibility. Provincial cancer registries in ON and NL were used to identify and confirm case eligibility. The Ontario Familial Colorectal Cancer Registry (OFCCR) [23] was established in 1997 as one of six international sites in the consortium of Colon-Cooperative Family Registry, C-CFR. Eligible cases had a confirmed diagnosis for incident primary invasive colon or rectal cancer [pathology confirmed ICD-9 codes: 153.0-153.9, 154.1-154.3 and 154.8 (ON & NL); or ICD-10 codes: 18.0-18.7, 19.9, 20.9 (NL only)] between 1997 and 2000 (OFCCR) or between 1999 and 2003 (NFCCR).

Controls were selected from the target population and were frequency matched on sex and 5-year age groups in cases. In ON, potential controls were identified through publicly available databases (residential telephone listings, or municipal property assessment files) , while controls in NL were selected through random digit dialing. [24]. With the consents of participants, this self-report survey was mainly comprised of three components, a family history questionnaire (FHQ), a personal history questionnaire (PHQ), and a food frequency questionnaire (FFQ).

The FFQ administered in the ON portion of the study was the Hawaii FFQ, which has 188 food items and been validated against 24-hour recalls among a multi-ethnic Hawaiian/ Southern Californian population and commonly used in the United States. [25-29]. The FFQ administered in NL was a modified version of the ON questionnaire that had been developed specifically to account for the unique food consumption patterns in NL [30,31] The NL questionnaire is very similar to the FFQ used in ON, however some food items that are not commonly consumed as part of the NL diet were excluded, and some food items (such as pickled meat and moose meat) commonly consumed in NL but not included in the Hawaii FFQ were added to the questionnaire. The NL questionnaire consisted of 169 items organized into 11 categories and required participants to recall their eating habits from one year prior to their diagnosis.

The FHQ and PHQ included many multiple-choice questions with regards to personal characteristic information as well as other details concerned, including body weight and height, medical history, bowel screening history, diet, medication use, diet, physical activity, reproductive factors, alcohol and tobacco use and socio-demographic measures (education and income).

Of the original population, we have excluded those who did not provide reliable and sufficient information during the survey (n = 918). Participants with reported energy intakes in the upper or lower 2.5% of intake (n = 225) (in NL, < 925 kcal and > 4700 kcal for men, < 1100 kcal and > 4900 kcal for women; In ON, < 1040 kcal and > 5200 kcal for men, < 835 kcal and > 4100 kcal for women) were also excluded because their FFQs were considered to be unreliable. Cases with familial adenomatous polyposis (FAP) or an in-situ colon or rectal tumor were excluded (n = 34).

The study was approved by corresponding research ethics authorities at University of Toronto and Memorial University of Newfoundland [32,33].

### Statistical analyses

For descriptive purposes, data were stratified by case-control status.

Odd ratios (ORs) and 95% confidence intervals (CIs) were estimated to ascertain associations of total energy and macronutrient intakes with colorectal cancer risk by using the unconditional logistic regression model with the SAS statistical software (version 9.1 SAS Institute, Cary, NC, USA). Stratified analyses by province were performed first and the ORs for main exposure variables were similar. Consequently, pooled analyses were conducted and province was treated as a covariate. Intakes of macronutrients and alcohol were adjusted for total energy intake via the residual method of Willett [34] that was used to reduce potential bias due to differential over- or under-reporting of food intakes. Various types of macronutrient were entered in the models both as quintiles of distribution of study population and continuously.

Age, sex, body mass index, physical activity, family history of CRC, polyps, diabetes, history of colon screening procedure, cigarette smoking, alcohol drinking, education attainment, household income, marital status, regular use of medication and supplements, reported hormone replacement therapy (HRT, females only), dietary intakes, and province of residence were evaluated as potential confounders. Covariate inclusion was based on whether there was a 10% or greater alteration in the parameter coefficient of interest. Covariates that met this criterion were included in a model, and a backwards-stepwise procedure was performed to obtain the final model; each nutrient had a unique set of confounding variables. Tests for linear trend of the ORs were conducted by modeling the median value for each category as a continuous variable in the analyses. Statistical tests were two sided, and *p *values less than 0.05 were considered statistically significant.

## Results

Table 1 shows the distribution of CRC cases and controls according to age, sex, province of residence, BMI and other selected variables. The study sample included 1760 cases (488 from NL and 1272 from ON) and 2481 controls (651 from NL and 1830 from ON) with average response rates of 65.0% and 53.5% in cases and controls, respectively. Among all the participants, cases were slightly younger than controls, and 55.7% of the cases were aged 60 years or older. Cases and controls had similar sex distribution. However, cases were more likely to be obese, to be either physically inactive or extremely physically active, and to have family history of CRC. Controls more often reported regular use of NSAID, higher education and income, and more frequent colon screening procedures.

**Table 1:** Selected characteristics of subjects from CRC case-control study in NL and ON

Characteristics^a^	Cases(n = 1760)	Controls(n = 2481)
	
	No. (%)	No. (%)
Age (years)*		
18-49	368(20.9)	265(10.7)
50-59	412(23.4)	690(27.8)
60-69	646(36.7)	998(40.2)
70+	334(19.0)	528(21.3)
Sex		
Males	935(53.1)	1357(54.7)
Females	825(46.9)	1124(45.3)
Province of residence		
NL	488(27.7)	651(26.2)
ON	1272(72.3)	1830(73.8)
BMI^b ^(kg/m^2^)*		
Underweight(< 18.5)	23(1.3)	22(0.9)
Normal(18.5-24.9)	595(33.8)	930(37.5)
Overweight (25-29.9)	748(42.5)	1069(43.1)
Obese(≥ 30)	394(22.4)	460(18.5)
Physical activity (METs/week^b^)*		
0 - 7.4	465(26.4)	595(24.0)
7.4 - 22.4	348(19.8)	633(25.5)
22.4 - 53.0	429(24.4)	633(25.5)
> 53.0	518(29.4)	620(25.0)
Family history of CRC*		
No	1582(89.9)	2337(94.2)
Yes	178(10.1)	144(5.8)
Reported any colon screening procedure*		
No	1500(85.2)	1861(75.0)
Yes	260(14.8)	620(25.0)
Regular use of NSAID^b^*		
No	1163(66.1)	1439(58.0)
Yes	597(33.9)	1042(42.0)
Education attainment*		
High school graduate or less	884(50.2)	1042(42.0)
Technical school/some college/university	540(30.7)	866(34.9)
Bachelor's degree/graduate degree	336(19.1)	573(23.1)
Household income ($CAN)*		
< 12,000	109(6.2)	154(6.2)
12,000-29,999	507(28.8)	573(23.1)
30,000-49,999	547(31.1)	777(31.3)
≥ 50,000	597(33.9)	977(39.4)

^a ^All characteristic variables presented as number(%).

^b ^BMI, body mass index; METs/week, metabolic equivalent hours per week; NSAID, nonsteroid anti-inflammatory drug.

* Significant differences between cases and controls (*p ≤ *0.05)

The mean daily intakes of total energy, macronutrients and alcohol among cases and controls are shown in Table 2. Cases reported significantly higher intakes of total energy, percentage of calories from total fat, percentage of calories from saturated fat and cholesterol (all *p *< 0.05) than controls. Controls had higher intakes of carbohydrate and total dietary fibre compared with cases (all *p *< 0.05).

**Table 2:** Mean intakes of total energy, macronutrients, and alcohol among subjects from CRC case-control study in NL and ON

Intakes of total energy and macronutrients^a^	Cases (n = 1760)	Controls (n = 2481)	Difference (Cases-Controls)
Total energy (kcal/day)*	2316.1 ± 810.6	2195.1 ± 750.8	121
Macronutrients			
Protein (g/day)	86.2 ± 18.5	87.2 ± 17.1	-1
% of Calories from Protein	15.2 ± 2.8	15.4 ± 2.9	-0.2
Carbohydrate (g/day)*	282.0 ± 49.7	286.2 ± 49.6	-4.2
% of Calories from Carbohydrates	49.6 ± 7.7	50.0 ± 8.0	-0.4
Total Fat (g/day)	81.3 ± 18.4	80.4 ± 18.0	0.9
% of Calories from Total Fat*	31.8 ± 6.1	31.3 ± 6.3	0.5
Dietary fibre (g/day)*	24.0 ± 8.5	25.2 ± 9.0	-1.2
Fatty Acids and Cholesterol			
Saturated Fatty Acids (g/day)	27.1 ± 7.0	26.8 ± 7.1	0.3
% of Calories from Saturated Fat*	10.6 ± 2.4	10.4 ± 2.6	0.2
Monounsaturated Fatty Acids (g/day)	29.6 ± 7.4	29.1 ± 7.2	0.5
Polyunsaturated Fatty Acids (g/day)	16.7 ± 5.0	16.6 ± 4.6	0.1
Cholesterol (mg/day)*	286.2 ± 116.4	277.1 ± 100.9	9.1
Alcohol (g/day)	7.4 ± 49.4	6.5 ± 36.8	0.9
% of Calories from Alcohol	3.9 ± 6.3	3.8 ± 5.9	0.1

^a ^All continuous variables presented as mean ± SD (standard deviation).

* Significant differences between cases and controls (*p ≤ *0.05)

Table 3 gives the ORs and corresponding 95% CI of CRC according to quintile intakes of macronutrients associated food components. After adjusting potential confounders, a significant increased risk of CRC was observed with increasing total energy intake (OR = 1.56 in the highest versus the lowest quintile of intake; 95% CI: 1.21-2.01), which the trend test was also significant (*p-*trend = 0.02). CRC risk was inversely related to intakes of carbohydrate (OR = 0.81, 95%CI: 0.63-1.00, *p-*trend = 0.05) and total dietary fibre (OR = 0.84, 95% CI: 0.67-0.99, *p-*trend = 0.04), both of them shown significant results in trend tests. An inverse association also emerged for intake of protein (OR = 0.85, 95%CI: 0.69-1.00), but the trend test was not significant (*p-*trend = 0.06). Neither association analysis nor trend test was significant for total fat, alcohol, saturated fatty acids, monounsaturated fatty acids, polyunsaturated fatty acids, cholesterol. We also evaluated percentage of kilocalories from macronutrients in relation to the risk of CRC and obtained similar results (data not shown).

**Table 3:** Associations (adjusted OR, 95%CI) of total energy, macronutrients, and alcohol intakes with CRC risk, CRC case-control study in NL and ON

Intakes of total energy, macronutrients, and alcohol	Quintiles of intakes			*p-*trend^b^
		
	Q1^e^	Q3^e^	Q5^e^	
Total energy				
No. of cases/controls	313/537	343/505	404/443	
Median intake (kcal/day)	1348.5	2109.3	3308.9	
OR^a ^(95% CI)	1.00	1.19(0.92,1.53)	1.56*(1.21,2.01)	0.02
Protein				
No. of cases/controls	372/479	334/513	335/512	
Median intake (g/day)	68.4	85.6	106.7	
OR^a ^(95% CI)	1.00	0.88(0.70,1.11)	0.85*(0.69,1.00)	0.06
Carbohydrate				
No. of cases/controls	392/458	332/516	334/513	
Median intake (g/day)	229.1	282.6	341.5	
OR^a ^(95% CI)	1.00	0.84(0.67,1.02)	0.81*(0.63,1.00)	0.05
Total Fat				
No. of cases/controls	344/506	362/487	372/475	
Median intake (g/day)	60.1	80.5	102.8	
OR^a ^(95% CI)	1.00	1.18(0.94,1.50)	0.96(0.75,1.22)	0.71
Total dietary fibre				
No. of cases/controls	388/462	355/493	308/539	
Median intake (g/day)	15.1	23.7	35.2	
OR^a ^(95% CI)	1.00	0.97(0.77,1.23)	0.84*(0.67,0.99)	0.04
Saturated Fatty Acids				
No. of cases/controls	346/504	331/517	378/469	
Median intake (g/day)	19.0	26.6	35.2	
OR^a ^(95% CI)	1.00	1.03(0.81,1.31)	1.00(0.79,1.26)	0.80
Monounsaturated Fatty Acids				
No. of cases/controls	341/509	342/506	371/476	
Median intake (g/day)	21.2	29.1	38.2	
OR^a ^(95% CI)	1.00	1.07(0.84,1.35)	0.99(0.78,1.26)	0.70
Polyunsaturated Fatty Acids				
No. of cases/controls	357/493	343/505	372/475	
Median intake (g/day)	11.6	16.4	22.4	
OR^a ^(95% CI)	1.00	1.03(0.81,1.30)	0.98(0.77,1.23)	0.47
Cholesterol				
No. of cases/controls	342/508	339/509	380/467	
Median intake (mg/day)	178.8	265.8	392.0	
OR^a ^(95% CI)	1.00	0.84(0.65,1.07)	1.00(0.79,1.28)	0.88
Alcohol				
No. of cases/controls	382/467	309/539	344/503	
Median intake (g/day)	0	13.6	182.8	
OR^a ^(95% CI)	1.00	0.88(0.67,1.17)	1.17(0.85,1.61)	0.38

^a ^Adjusted for total energy intake. Other potential confounders included age, sex, BMI, physical activity (METs/week), family history of CRC, polyps, diabetes, reported colon screening procedure, cigarette smoking, alcohol drinking, education attainment, household income, marital status, regular use of NSAID, regular use of multivitamin supplements, regular use of folate supplement, regular use of calcium supplement, reported HRT (females only), province of residence, and intakes of fruits, vegetables, and red meat. Variables were included in the final model based on a ≥ 10% alternation in the parameter coefficient of interest.

^b ^Two-sided *p *value for test of linear trend was calculated by using median values for each quintile of intake.

^e ^Q1 for quintile 1, Q3 for quintile 3, Q5 for quintile

* Significant different from reference category, *p *≤ 0.05

The relationship between intakes of total energy, protein, carbohydrate and dietary fibre with CRC risk was further examined in strata of various covariates. No substantial heterogeneity was observed in separate strata of sex; age(≤ 60, > 60 years); BMI(< 25, ≥ 25 kg/m2); physical activity(< 22.4, ≥ 22.4 METs/week); family history of CRC(no, yes); reported colon screening procedure(no, yes); NSAID use(no, yes); education attainment(lower, higher); household income(lower, higher), and total energy intake(≤ 2109.3, > 2109.3 kcal/day) (data not shown).

## Discussion

Our present case-control study, rather large among investigations of diet and CRC to-date, showed that intakes of total energy were significantly positively associated with risk of CRC, whereas inverse associations were seen with intakes of protein, carbohydrate, and dietary fibre. Intakes of total fat, fatty acids, cholesterol and alcohol were unrelated to the risk.

Our study observed a direct association between total energy intake and the risk of CRC, confirming results from several previous studies of other populations [11,15,35-37]. Studies of potential mechanisms revealed that revealed the evidence that caloric restriction reduces cancer incidence in rodents [38,39] as well as colorectal cell proliferation in humans [40-42]. Our study found that CRC risk was positively related to percentage of calories consumed as alcohol but was not related with energy-adjusted total alcohol intake. These results suggest that alcohol, independent of its contribution to energy, may not be associated with CRC risk. Energy intake can be responsible for glycemic overload and a compensatory increase of serum insulin and related insulin growth factor-1 (IGF-1). IGF-1 is a promoter of tumor cell growth in vitro [43,44], and it may expose colonic and rectal cells to a proliferative stimulus [45,46]. Diabetes has also been related to increased CRC risk [47-49]. Additionally, higher energy intake, in the absence of compensatory energy expenditure leads to excess body weight, which in-turn, is an established risk factor for CRC (Campbell et al., 2007 CEBP; Campbell et al., 2010, JNCI, both studies used these data).

Williams *et al. *[14] found that the risk of CRC moderately decreased with an increase of protein intake (OR = 0.53, 95% CI = 0.37-0.77). Consistent with Williams' results, findings of our study showed that CRC risk was inversely associated with protein intake (OR = 0.85, 95%CI: 0.69-1.00) and percentage of calories from protein intake (OR = 0.76, 95%CI: 0.61-0.96). In all kinds of food, meat, fish, soy and eggs were the main sources of protein intake. In our study population, red meat intake was moderate (about 4 servings/week) with similar proportions (approximately 20%) of proteins derived from red meat, dairy products and the combination of white meat and fish. Meta-analyses of meat consumption and CRC risk have concluded that red meat and processed meat increase the risk of CRC [50] and that processed meat may be a stronger risk factor than fresh red meat [51,52]. Thus, non-red meat sources of animal protein may have a beneficial influence [18]. Moreover, several previous studies have consistently found inverse associations with high protein foods (dairy products, white meat, fish and poultry) or with non-red meat protein [14,53,54]. In our study, a clearer inverse association with protein may have emerged if non-red meat protein sources were analyzed separately from red meat. A possible explanation for a protective effect of protein is that low intakes of methionine may contribute to DNA methylation abnormalities, which appear to be important in the initiation and progression of colon cancer [55].

Carbohydrate intake was shown to be inversely related to CRC risk in our study. Compared with participants in the lowest quintile of carbohydrate consumption, those in the highest quintile were 19% less likely to develop CRC. There is limited and inconsistent epidemiologic evidence for a relationship between carbohydrate intake and CRC risk. A study conducted among Chinese in North America found that total carbohydrate consumption was associated with increased risk of CRC [56], whereas a survival analysis reported high carbohydrate was strongly elevate survival of CRC [57].

We also observed an inverse association with fibre intake, which was in agreement with several previous studies [15,53,58,59]. In our study, a 16% reduced risk of CRC was observed among participants with higher intake of fibre. A beneficial effect from fibre may arise by several mechanisms, including accelerated the fecal transit time, dilution of colonic contents. [12].

We found no evidence of any substantial effect of the intake of total fat, saturated fatty acids, monounsaturated fatty acids, polyunsaturated fatty acids or cholesterol on risk of CRC in the present study. These results are consistent with most previous studies [14,15,18,60], but not all [16]. However, our finding again suggests that fat, independent of its contribution to energy, may not be associated with CRC risk. Although there exists a sensible biological rationale for the possible involvement of fat in colorectal carcinogenesis [61], it appears that if fat is indeed involved, the mechanism must be more complex than that which would be implied by a simple empirical association with daily fat intake per day. It could involve foods or some complex interaction amongst nutrients or other food components. Discussion of such potential biological mechanisms is beyond the scope of the present paper.

Consideration must be given to the potential limitations in the present study that may have influenced the observed associations. Firstly, as in most case-control studies, potential recall and selection biases are possible. Since exposure information was collected after diagnosis, differential recall between cases and controls would bias results; in particular, cases may recall dietary exposures differently from controls because of the presence of illness or symptoms. Controls may have agreed to join this study because of an interest in health and may therefore have healthier dietary and physical activity habits, a pattern that may exaggerate differences with the cases beyond what might have been seen with truly comparable controls.

Secondly, by design, cases and controls had similar sex distributions; however, even though analyses were age-adjusted, it may be noted that cases and controls were not well matched according to age group. Estimates of nutrient intakes from a FFQ are not precise and there is always the potential for measurement error. Although the original FFQ used in this study has been validated [28,29], this questionnaire requires further evaluation because it was originally developed for the Hawaiian and Californian populations which may be different from people residing in NL and ON. FFQ used in NL has been adapted to include regional foods in NL; however, OFCCR used the original FFQ that has not been adapted. Thus, a sub-study will be necessary to assess the level of agreement between the FFQ used by the OFCCR and the FFQ that was previously developed specifically for Canadian populations. Finally, it is also possible that the 1-2 year referent period on which dietary data were based is insufficient if more remote diet (eg. 5-10 yrs) has a stronger influence on CRC risk.

This study had a number of strengths. The large sample size allowed us to observe associations that might be undetectable in smaller studies. More importantly, the previous findings about the protective effects of macronutrients were confined to a specific study population, which makes it difficult to generalize the results. In this study, we conducted pooled analyses of the population of two Canadian provinces to investigate the associations of total energy, macronutrients, alcohol and CRC risk. Furthermore, nutrient intakes were adjusted for total energy intake. The use of calorie-adjusted values in multivariate models will often overcome the problem of high co-linearity frequently observed between nutritional factors [34]. To the extent that, this adjustment also reduces between-person variation due to over- or underreporting of food intakes [34]. The relationships of total energy, macronutrients, alcohol and CRC risk may differ appreciably by several factors, so we controlled for a wide range of potential confounding factors using multivariate logistic regression models. Additionally, results of the consistent findings in separate strata for total energy, protein, carbohydrate and dietary fibre would argue against multiple comparisons as an explanation for these associations. Although some random misclassification of diet is likely, non-differential misclassification generally tends to bias the risk estimates toward the null.

## Conclusion

In conclusion, findings of our large population-based case-control study of CRC conducted in two Canadian provinces provide further evidence that diets high in energy may increase the risk, whereas diets high in protein, fibre, and carbohydrate may reduce the risk of CRC. These results underline the importance of some aspects of total energy and macronutrients and consequently the potential for prevention through dietary changes.

## Abbreviations

CRC: colorectal cancer; NL: Newfoundland and Labrador; ON: Ontario; NFCCR: Newfoundland Familial Colorectal Cancer Registry; OFCCR: Ontario Familial Colorectal Cancer Registry; C-CFR: Colon-Cooperative Family Registry; ICD: international classification of disease; FHQ: family history questionnaire; PHQ: personal history questionnaire; FFQ: food frequency questionnaire; NSAID: nonsteroidal anti-inflammatory drug; BMI: body mass index; HRT: hormone replacement therapy; METs: metabolic equivalent hours; RDA: recommended dietary allowances; OR: odds ratios; 95%CI: 95% confidence interval.

## Conflicts of interests

The authors declare that they have no competing interests.

## Authors' contributions

The work presented here was carried out in collaboration between all authors. ZS, the principal investigator, performed the data analyses and drafted the manuscript; LL conducted the literature search and revision of this article; PPW conceived of the study, participated in its conceptualization, design and coordination. JZ, SB, BR, ED, JRM, PTC, PSP, the co-project investigators, contributed to the execution of this study at various stages. All authors read and approved the final manuscript.
